# LiDAR Intensity Completion: Fully Exploiting the Message from LiDAR Sensors

**DOI:** 10.3390/s22197533

**Published:** 2022-10-04

**Authors:** Weichen Dai, Shenzhou Chen, Zhaoyang Huang, Yan Xu, Da Kong

**Affiliations:** 1 School of Computer Science, Hangzhou Dianzi University, Hangzhou 310018, China; 2 State Key Laboratory of Industrial Control Technology, College of Control Science and Engineering, Zhejiang University, Hangzhou 310007, China; 3 Alibaba A.I. Labs, Hangzhou 311121, China; 4 Electronic Engineering, Chinese University of Hong Kong, Hong Kong 999077, China

**Keywords:** LiDAR intensity completion, LiDAR sensors, intensity normalization

## Abstract

Light Detection and Ranging (LiDAR) systems are novel sensors that provide robust distance and reflection strength by active pulsed laser beams. They have significant advantages over visual cameras by providing active depth and intensity measurements that are robust to ambient illumination. However, the systemsstill pay limited attention to intensity measurements since the output intensity maps of LiDAR sensors are different from conventional cameras and are too sparse. In this work, we propose exploiting the information from both intensity and depth measurements simultaneously to complete the LiDAR intensity maps. With the completed intensity maps, mature computer vision techniques can work well on the LiDAR data without any specific adjustment. We propose an end-to-end convolutional neural network named LiDAR-Net to jointly complete the sparse intensity and depth measurements by exploiting their correlations. For network training, an intensity fusion method is proposed to generate the ground truth. Experiment results indicate that intensity–depth fusion can benefit the task and improve performance. We further apply an off-the-shelf object (lane) segmentation algorithm to the completed intensity maps, which delivers consistent robust to ambient illumination performance. We believe that the intensity completion method allows LiDAR sensors to cope with a broader range of practice applications.

## 1. Introduction

Unlike visual cameras, which passively capture the light emitted or reflected by objects, LiDAR sensors actively project pulsed laser beams and measure the surrounding environment through backscattered echoes [1]. Therefore, LiDAR sensors can function well even in adverse illumination conditions. With robust depth measurements, LiDAR sensors are crucial to many applications such as autonomous vehicles [2,3], classification [4], and instance detection [5,6].

The intensity output of LiDAR sensors is a stream of laser reflection. With this unique mechanism, mobile LiDAR sensors such as Velodyne VLP-16 can provide richer object material information in addition to the depth map. However, the unique mechanism only provides intensity measurements with sparse circular results. It leads to the situation that existing computer vision techniques, including optical flow [7], relocalization [8], and object (lane) segmentation [9], all having demonstrated impressive performance on visual images, cannot directly operate on this unique sparse LiDAR intensity structure.

To acquire dense intensity maps from LiDAR sensors, some works focus on adding a more complicated rotation mechanism, such as two-axis scanning with one laser sensor [10], to obtain a dense intensity map, called LiDAR-intensity imagery [11]. Based on this dense map, visual-liked navigation [12] and perception [13] can be produced on the LiDAR intensity. The shortcoming is that there is a distortion due to motion and the LiDAR’s scanning nature, akin to a slow-rolling shutter camera, and the distortion is hardly compensated. Additionally, since the rotation mechanism rotates slowly, many regions will be missed when the carrier moves too fast. Another solution is to increase the number of lasers as much as possible, such as VLS-128. Nevertheless, it still cannot provide the image-level density and inevitably leads to unacceptable prices.

**Figure 1:**
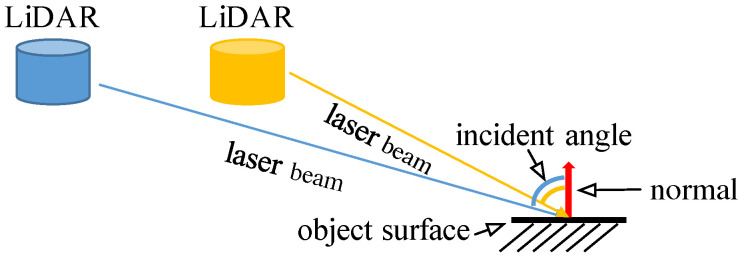
Intensity measurements. The attenuation in the traveling and the incident angle can influence the received intensity. Therefore, from different views, the intensity values are different for the same position.

It should be noted that the sparse depth measurements also limit the performance in handling tasks such as object detection and navigation. Hence, various works focus on filling in the missing depth values on a dense depth map using only depth measurements, and this is called depth completion [14,15,16,17,18]. Following this thought, LiDAR intensity completion may be an complementary solution. As far as we know, very few methods have been proposed to address the LiDAR intensity completion. Current public completion-related LiDAR datasets, such as KITTI Completion Benchmark [19], do not provide the benchmark for LiDAR intensity completion, and hence the evaluation is still in the preliminary stage. Meanwhile, compared to the depth, the intensity is much more complicated. As shown in Figure 1, the intensity measured by LiDAR can be decomposed into four main factors under the Lambertian reflectance assumption [20]: measurement distance, the surface reflectance, the strength of the incident ray, and the incident angle [21]. These factors make the LiDAR intensity completion more challenging. It should be noted that the measurement distance is one of the impact factors, implying that the intensity is correlated with the measurement distance.

In this paper, we propose a LiDAR intensity completion method to bridge the gap between LiDAR intensity and conventional computer vision. For the LiDAR intensity completion, we propose LiDAR-Net, a convolutional neural network to simultaneously learn both intensity and depth information from LiDAR sensors to complete the intensity map. LiDAR-Net consists of an encoder–decoder network with LiDAR information fusion and an inverse network. The encoder consists of a sequence of convolutions to downsample the resolution. The decoder, on the other hand, has a reversed structure with transposed convolutions to upsample the resolution. Between the contracting and symmetric expanding paths, long skip connections improve the sharpness of completion results. A network with several convolutions produces material-related intensity maps using the results of the encoder–decoder network.

To train this network, a vehicle carrying a Velodyne HDL64 LiDAR was used to collect data in two 500 × 500 $m^{2}$ areas. Then, we rectified the raw intensity by Lambertian reflection model and fused sparse intensity maps from a sequence of data frames to build an intensity dataset. To further validate the potential of dense completed intensity maps, an object segmentation method was used to detect the lanes on the road.

The main contributions of this work can be summarized as follows:

- LiDAR-Net, a novel intensity completion method is proposed using intensity–depth fusion. The experiment results show that the proposed method can provide competitive performance compared with state-of-the-art completion methods.
- A LiDAR intensity fusion method is proposed to generate the intensity ground truth for training. Using multiple types of intensity data from the proposed method for training can improve the performance of the LiDAR intensity completion.
- The proposed method is tested in object (lane) segmentation based on completed intensity maps. The result shows that off-the-shelf computer vision techniques can operate on the completed LiDAR intensity maps. Moreover, the LiDAR intensity completion provides more robust lane segmentation results than visual cameras under adverse conditions.

The rest of the paper is organized as follows. Section 2 reviews related work. Section 3 first introduces the proposed intensity fusion method for ground truth generation, then discusses the proposed architecture and how to train the network on the self-built dataset. Furthermore, Section 4 presents the experimental results in detail. Finally, conclusions are drawn in Section 5.

## 2. Related Work

There are two types of perception technologies: passive and active, according to the energy source used in the detection. LiDAR sensors and visual cameras are representative of these two classes, respectively. Since LiDAR sensors function well even in adverse lighting conditions, they are crucial to many applications. In these applications, 3D object detection is an important task [22,23,24,25,26]. However, for those objects without spatial volumes, such as road signs on the ground, they cannot be detected using the point cloud data. Therefore, the intensity from LiDAR can be a valuable information source.

### 2.1. LiDAR Intensity

**Figure 2:**
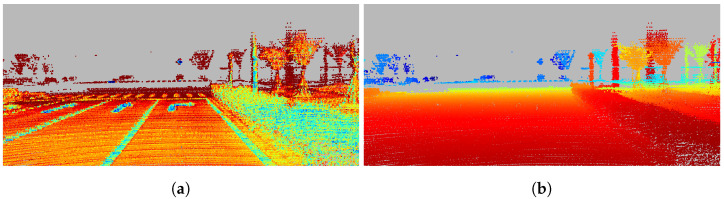
Densified intensity and depth maps obtained from multiple frames: (**a**) densified intensity (**b**) densified depth.

LiDAR intensity measurements have implicit correlations with depth measurements, as shown in Figure 2. Moreover, LiDAR intensity is steadily available even in some adverse conditions since LiDAR sensors are robust to lighting variation [27]. Therefore, the intensity measurements are a potential information source. Nonetheless, intensity measurements are dependent on multiple factors and, therefore, difficult to model. The surface observed by the sensors is assumed not to contain mixed microstructures and conforms to the Lambertian reflectance assumption [20]. In that case, the factors affecting the intensity strength measured by LiDAR consist of three main parts: the surface reflectance, the strength of the incident ray, and the incidence angle.

Following the Lambertian assumptions, the researchers proposed corresponding theoretical methods to correct geometric effects [28,29,30]. However, the theoretical model parameters are difficult to compute accurately and are challenging to apply to short distances (e.g., 10 m) [27]. Hence, Höfle and Pfeifer adopted an empirical quadratic function related to the depth to correct intensity [31]. Moreover, refs. [28,32] normalized the intensity to obtain the intensity only related to the surface reflectivity. The limitation is that these methods require dense depth maps, which prohibits their adaptation to sparse scanners such as vehicle-mounted LiDAR.

### 2.2. Sparse to Dense

Intensity information contains much valuable information. Therefore, some work has focused on using intensity to accomplish tasks [1]. Aided by intensity information, ref. [1] presents some examples: [33] detected the damage and degradation of concrete structures, ref. [34] detected road markings and maintenance holes, etc. However, most of these works rely on the dense intensity maps, which the LiDAR mounted on a moving car usually cannot directly provide.

To obtain dense intensity maps, two approaches were proposed: hardware-based and algorithm-based methods. The hardware-based method ensures that the laser traverses the entire area by adding a complex rotating mechanism [12]. The algorithm-based method uses the correlation between discrete sample points to estimate the missing information. Asvadi [35] proposed using Delaunay triangulation to interpolate the missing intensity. Melotti [36] generated dense depth and intensity maps through a bilateral filter implementation, which was used as input to the CNN network to achieve pedestrian classification. These methods need neighbor information to interpolate the missing data, which requires sufficient information near the interpolated point. This requirement limits the application of these methods to further sparse structures.

The algorithm-based LiDAR intensity completion methods are similar to the methods used for depth completion [17,18,37,38]. These depth completion methods employ hand-crafted features or kernels to complete the missing values. Most methods are only designed explicitly for Kinect sensors that inherently provide more dense depth maps with different techniques than LiDAR. Recently, the learning-based approaches showcase their promising performance thanks to the rapid advance of deep learning. Uhrig [19] creatively proposed enhancing the sparse LiDAR depth measurements via the sparsity-invariant convolution layer. Moreover, Eldesokey [39] modeled confidence propagation through layers to reduce the number of model parameters.

The works mentioned above accomplished the depth completion only from the depth information. However, it was soon found that the information from other modalities, e.g., color images, can significantly improve the performance [14]. Recently, more explorations in network design have been conducted to harness deep neural networks’ power [15,16,40,41,42,43]. Nevertheless, none of these methods can be directly applied to LiDAR intensity completion. Compared to depth completion, the case for LiDAR intensity is more complicated since the intensity is determined by the incidence angle, surface reflectance, and distance from the sensor, making it difficult for the network to grasp the essence with limited training data.

## 3. Method

This section first introduces the proposed intensity fusion method to generate densified intensity ground truth since the raw LiDAR measurement is too sparse. After that, the LiDAR-Net, a novel supervised neural network that completes depth and intensity simultaneously, will be introduced.

### 3.1. Intensity Fusion for Ground-Truth Generation

Since the LiDAR sensor is not motionless, each data frame is obtained from a different view. The challenge is that raw intensity describes the reflected pulse’s strength, which is inconsistent under different views. This leads to different intensity values from the same position with different measurement distances or angles. Hence, a dense intensity map cannot be directly generated using adjacent frames.

**Figure 3:**
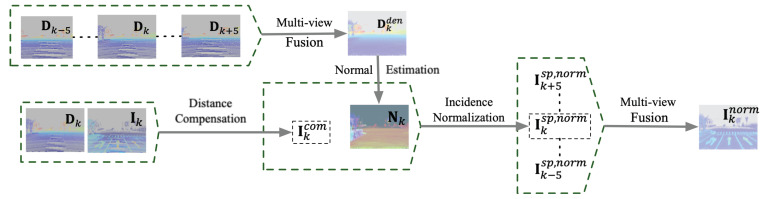
Distance compensation, incidence normalization, and multi-view fusion. The pixels on the image are enlarged five times to increase the visualization. The raw intensity measurement is compensated by the corresponding depth map and merged by poses to obtain $I_{k}^{com}$. The incident angle is obtained from $N_{k}$ of the fused depth map to avoid the error caused by the normal estimation in the sparse point cloud. The multiple normalized intensity maps are fused to obtain a densified one.

**Figure 4:**
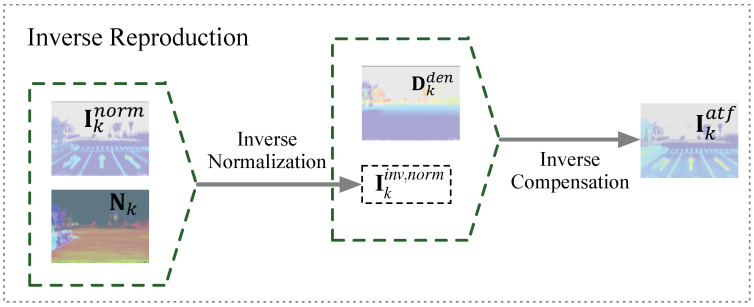
Inverse reproduction. The densified $I_{k}^{norm}$ will be inversed to produce more dense artificial intensity maps. This process is the inverse process of the above steps except for the multi-view fusion.

As mentioned in Section 2, the intensity values are mainly related to the distance traveled by light, the surface reflectivity, and the incident angle. Only the reflectance of the object surface is consistent. Therefore, we need to eliminate the effect of distance and incident angle before fusing multi-view data. In summary, as shown in Figure 3 and Figure 4, the intensity fusion method consists of four main steps to obtain the intensity ground truth: distance compensation, incidence normalization, multi-view fusion, and inverse reproduction.

Before introducing the intensity fusion method, some notations will be defined first. The depth and intensity maps collected at the sampling time *k* by projecting LiDAR sensor data are denoted by $D_{k}$ and $I_{k}$, where $D_{k},I_{k}:R^{2}\mapsto R$.

#### 3.1.1. Distance Compensation

The compensated intensity map $I_{k}^{com}$ is computed from the raw intensity map $I_{k}$ through

(1) $$I_{k}^{com}\left(u\right)=I_{k}\left(u\right)+g\left(D_{k}\left(u\right)\right),$$

where $u\in R^{2}$ is a map position vector, and g(·) is a distance-aware compensation term defined by

(2) $$g\left(D\left(u\right)\right)=focal+K\left(1-\frac{D_{k}{\left(u\right)}^{2}}{{d^{ref}}^{2}}\right),$$

where *K*, focal, and $d^{ref}$ are the intrinsic parameters of each laser beam after the official calibration [44]. The compensated intensity values can thereby be almost independent of depth.

#### 3.1.2. Incidence Normalization

The incidence normalization step will normalize $I_{k}^{com}$ to obtain the normalized intensity map, which is irrelevant to the incident angle. First, it is assumed that all surfaces follow the Lambertian Cosine Law [20]. With this assumption, the compensated intensity $I_{k}^{com}$ can be normalized into a sparse normalized intensity $I_{k}^{sp,norm}$ by:

(3) $$I_{k}^{sp,norm}\left(u\right)=I_{k}^{com}\left(u\right)\left(\frac{1}{\cos(v)}\right)/\eta ,$$

where *v* is the incident angle corresponding to the position u, and η is a constant coefficient that allows the normalized intensity to be distributed between 0 and 255. In the implementation, a data-driven parameter estimation method [32] is applied to estimate the parameters and obtain the optimal parameter value η=5.05 in Equation (3).

The only missing parameter is the incident angle *v*, which can be calculated with the depth map. However, the LiDAR sensors only provide sparse depth measurements, which cannot produce accurate surface normal estimation. Hence, the previous and subsequent LiDAR data frames are reprojected to the current frame to obtain a more dense depth map $D_{k}^{den}$,

(4) $$\begin{array}{cc} {\left[u^{\prime T},d\right]}^{T} & =\pi (T_{k,j}\pi^{-1}\left(u,D_{j}\left(u\right)\right))\\ D_{k}^{den}\left(u^{\prime }\right) & =d \end{array},$$

where $T_{j,k}$ denotes the transformation between the *k*-th and the *j*-th frame, and $\pi_{a}:R^{3}\mapsto R^{3}$ is the projection model to obtain the map position and the corresponding depth value. Thus, with ground-truth poses, a densified depth map is collected by using multiple frames. In the implementation, 11 frames are projected onto the current frame according to the known transformation matrix.

With the densified depth map, the surface normal will be estimated so that cosv can be computed by

(5) cos(v)=<L,N>

where L is the incidence light direction, and N is the surface normal. With cos(v), the normalization can be used to recover $I_{k}^{sp,norm}$ from $I_{k}^{com}$.

#### 3.1.3. Multi-View Fusion

Since $I_{k}^{sp,norm}$ is irrelevant to the depth and incidence angle, similar to the depth map re-projection mentioned in incidence normalization, a more dense $I_{k}^{norm}$ will be acquired through reprojection of the multi-view normalized intensity maps. The missing value will be filled by

(6) $$\begin{array}{cc} {\left[u^{\prime T},d\right]}^{T} & =\pi (T_{k,j}\pi^{-1}\left(u,D_{j}^{den}\left(u\right)\right))\\ I_{k}^{norm}\left(u^{\prime }\right) & =I_{j}^{sp,norm}\left(u\right) \end{array},$$

where j=k−5,k−4,⋯,k+4,k+5.

#### 3.1.4. Inverse Reproduction

This operation consists of inverse normalization and inverse compensation. These two steps can be considered as the inverse version of the incidence normalization and distance compensation using dense data. In this operation, we will inverse the densified $I_{k}^{norm}$ to generate the artificial raw intensity $I_{k}^{atf}$ according to

(7) $$\begin{array}{cc} I_{k}^{inv,norm}\left(u\right) & =I_{k}^{norm}\left(u\right)\cos\left(v\right)\eta \\ I_{k}^{atf}\left(u\right) & =I_{k}^{norm}\left(u\right)-g(D_{k}^{den}\left(u\right))\prime \end{array}$$

where $D_{k}^{den}$ and cos(v) were obtained from the previous steps. An example is shown in Figure 4.

### 3.2. LiDAR-Net

**Figure 5:**
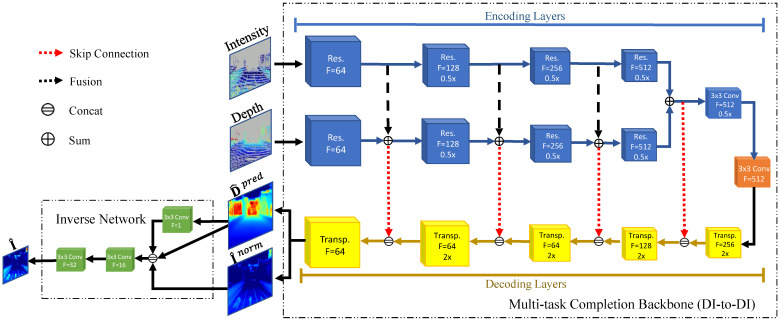
LiDAR-Net architecture. The pixels on the input are enlarged five times to increase the visualization. The output ${\hat{D}}^{pred}$ and ${\hat{I}}^{norm}$ of the backbone will be input for the inverse normalization network, as shown in the upper left corner. Because it is difficult to quickly estimate the normal using ${\hat{D}}^{pred}$, the inverse normalization network attempts to fit the inverse normalization process shown in Equation (7) with several convolution layers and predicts the dense $\hat{I}$ supervised by $I^{atf}$.

As shown in Figure 5, the LiDAR-Net, including the multi-task completion backbone and the inverse normalization parts, completes the intensity using intensity and depth measurements simultaneously.

The multi-task completion backbone consists of a contracting path(encoding layers) to capture the shared context and a symmetric expanding path(decoding layers) that enables precise prediction [45]. We fuse the features extracted from depth and intensity to combine geometry and reflectivity information in the contracting path. Meanwhile, high-resolution features from the contracting path are combined with the upsampled output. Therefore, this network can give a more precise prediction. Since the material-related $I^{norm}$ is more consistent under different views, it is used for training the backbone with the depth map.

In the inverse network, a successive network fuses the backbone’s output to provide a dense intensity. This network will be supervised by the artificial intensity $I^{atf}$. Finally, the LiDAR-Net will predict first ${\hat{I}}^{norm}$ and then $\hat{I}$.

#### 3.2.1. Architecture

The densified depth $D^{den}$ and $I^{norm}$ are the ground truth of the Multi-task Completion Backbone shown in Figure 5. The feature extraction (encoding) layers of the network are highlighted in blue. Both intensity and depth encoders consist of a series of ResNet blocks [46]. The sum of each layer’s depth and intensity features in the encoder is concatenated with the corresponding decoder layer. The last component of the encoding structure, a convolution layer, is used to further downsize the feature resolution. In the intermediate layer, a convolution shown in orange has a kernel size of 3-by-3. The decoding layers highlighted in yellow consist of five transposed convolutions to upsample the spatial resolution and combine the information from both intensity and depth encoders.

Since it is difficult to quickly estimate the normal without guidance, an inverse normalization network will be set to attempt to fit the inverse process with several convolutional layers and predict the dense ${\hat{I}}^{arf}$ supervised by $I^{arf}$. Therefore, after backbone processing, ${\hat{D}}^{pred}$ and ${\hat{I}}^{norm}$ will be input into the inverse normalization network, as shown in the upper left corner of Figure 5.

In the LiDAR-Net, all convolutions are followed by batch normalization [47] and ReLU, except at the last layer.

#### 3.2.2. Training

The training is divided into two steps. First, $I^{norm}$ and its corresponding dense $D^{den}$ are used to supervise the multi-task completion backbone. After the convergence, the entire LiDAR-Net, including the multi-task completion backbone and the inverse network, will be supervised by $I^{arf}$, $I^{norm}$, and $D^{den}$ to obtain the optimal intensity prediction. The difference between the network input and output is penalized on a pixel set of available known sparse depth. The depth loss is defined as

(8) $$L_{Depth}=\sum_{u,k}{∥{\hat{D}}_{k}^{pred}\left(u\right)-D_{k}^{den}\left(u\right)∥}_{2}.$$

Similarly, for normalized intensity maps, the loss through all available data can be defined as

(9) $$L_{I^{norm}}=\sum_{u,k}{∥{\hat{I}}_{k}^{norm}\left(u\right)-I_{k}^{norm}\left(u\right)∥}_{2}.$$

In summary, the $L_{2}$ loss is minimized in the training process, and the overall loss function containing two terms to supervise ${\hat{D}}^{pred}$ and ${\hat{I}}^{norm}$ is defined by

(10) $$L=L_{Depth}+\alpha L_{I^{norm}},$$

where α is a weighting coefficient, which is set to 0.3.

In the second step, the proposed network can simultaneously complete the depth, the material-related ${\hat{I}}^{norm}$, and the theoretical dense intensity value of the current frame $\hat{I}$. The loss for final intensity completion is defined as

(11) $$L_{I^{norm}}=\sum_{u,k}{∥{\hat{I}}_{k}\left(u\right)-I_{k}^{atf}\left(u\right)∥}_{2}.$$

In this step, the overall loss function contains three terms to supervise ${\hat{D}}^{pred}$, ${\hat{I}}^{norm}$, and $\hat{I}$, and is defined by

(12) $$L=L_{Depth}+\alpha L_{I^{norm}}+\beta L_{I^{arf}},$$

where α and β are weighting coefficients and are set to 0.3 and 0.02 in the experiments, respectively.

## 4. Experiments

In this section, the quality of the generated ground truth in the dataset is first evaluated. Then, the proposed LiDAR-Net network is evaluated in detail. Since there is no open-source method designed for LiDAR intensity completion and no open-source method using depth and intensity information for completion simultaneously, first we use depth completion methods for comparison and then design an ablation experiment to verify and demonstrate the proposed algorithm’s effectiveness and accuracy. To further illustrate the intensity–depth fusion’s effectiveness, we design a comparison experiment with the state-of-the-art depth completion algorithm, which only utilizes depth information. Finally, an off-the-shelf vision technique will operate on the dense intensity maps obtained from the proposed network to find the lanes on the road. This experiment will verify that LiDAR intensity completion can be used to bridge the gap between vision techniques and LiDAR sensors.

**Figure 6:**
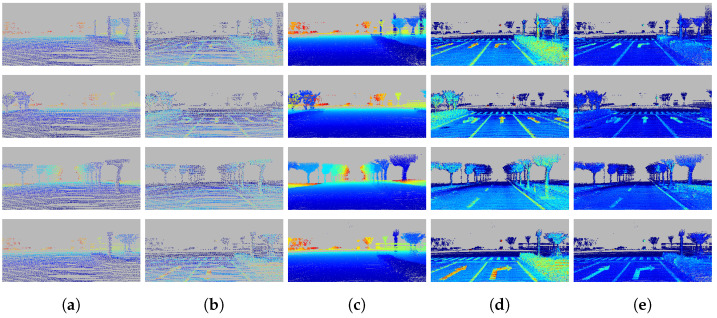
The overview of the dataset. Four different instances are demonstrated. Each pixel in (**a**,**b**) is enlarged five times to increase the visualization. Colder color in the depth map indicates a farther distance, while colder color in the intensity map indicates a weaker strength. The dataset consists of two inputs: sparse depth and sparse intensity. It has three ground-truth values: densified depth, artificial intensity $I^{atf}$, and normalized intensity $I^{norm}$. $I^{norm}$ eliminates the influence of distance and the incidence angle, resulting in sharper edges between different materials and a better consistency within the same material: (**a**) sparse input depth D; (**b**) sparse input intensity I; (**c**) densified depth $D^{den}$; (**d**) artificial intensity $I^{atf}$; (**e**) normalized intensity $I^{norm}$.

**Figure 7:**
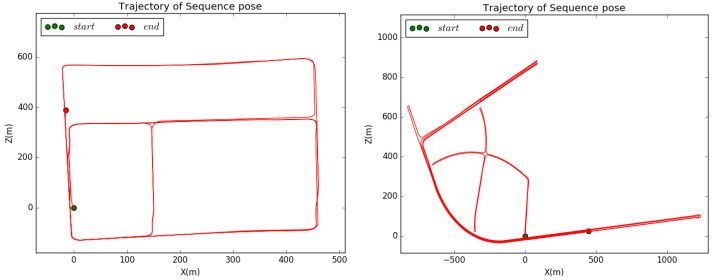
Trajectories of the two scenes in the dataset.

Since none of the existing datasets provide training data of dense depth and intensity for completion, we use the proposed intensity fusion method to create a dataset to obtain the ground truth for training and validation. As shown in Figure 6, there are two scenes. A vehicle carried a Velodyne HDL64 LiDAR and collected data for two 500 × 500 $m^{2}$ areas, as shown in Figure 7, at a speed of about 40 km per hour. A Global Navigation Satellite System (GNSS) called Novatel provided accurate pose information.

Our dataset takes the sparse depth and the measured raw intensity as input, and the densified depth maps, $D^{den}$, $I^{norm}$, and $I^{atf}$ as the ground truth. The train/validation/test sets contain 8647/1730/300 frames, and each frame is scaled from 1762×800 to 800×288. Moreover, to prove the generality of this method, a dataset was generated in different environments with more dynamic scenes in another city in the same way, and the train/validation/test sets contain 9340/1037/697 frames scaled to 800×288. Point clouds with intensity and depth from 11 frames are accumulated, as mentioned in Section 3.1, to increase density.

In the implementation, LiDAR-Net was trained using the Adam [48] optimizer with an initial learning rate of 0.0001 for 40 epochs with a batch size of 8. The learning rate was reduced to 10% every ten epochs, and the weights α and β were set to 0.3 and 0.02. We used four Nvidia GTX 1080Ti GPUs with 11Gb of RAM, and it took roughly 16 h to train the LiDAR-Net.

For each network, we tried our best to adjust the parameters and record the optimal values. The error metric of the KITTI depth completion benchmark, including the root mean square error (RMSE) and the mean absolute error (MAE), was used as the evaluation indicator.

### 4.1. Evaluation of Intensity Ground Truth

To evaluate the quality of the obtained ground truth, we analyzed each step’s effect in the intensity fusion method, firstly through qualitative perspectives and then by a numerical quantitative analysis, to illustrate some visual intuition and enhance the credibility.

The essential part of the proposed intensity fusion method is to obtain consistent intensity information from different viewing angles and different distances. Therefore, the intensity distribution of the same material in the normalized intensity maps should show consistency. After the inverse reproduction, the artificial intensity maps should have the same distribution as the raw intensity maps.

#### 4.1.1. Consistency in Normalized Intensity Maps

As mentioned in Section 3.1, we sequentially used Equations (1) and (3) to compensate and normalize each frame’s raw intensity map before the fusion. Since the obtained $I^{norm}$ should be only related to material reflectivity, the intensity values should be more consistent in the same material.

**Figure 8:**
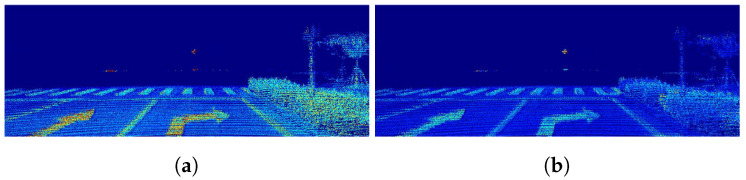
Two types of intensity ground truth (enhanced for visualization): (**a**) artificial intensity $I^{atf}$; (**b**) normalized intensity $I^{norm}$. The boundaries of different materials of $I^{norm}$ are much more distinguishable. In addition, the intensity of the same material shows consistency in $I^{norm}$. (**a**) $I^{atf}$. (**b**) $I^{norm}$.

As shown in Figure 8, the arrows on the road surface show that the normalized intensity $I^{norm}$ of the same material is more robust to variations in the distance and incidence angle than $I^{atf}$. Moreover, the $I^{norm}$ of the shrub remains consistent even when the incidence angle changes drastically. Furthermore, $I^{norm}$ shows an advantage in distinguishing between different materials, such as the lane and the road.

**Figure 9:**
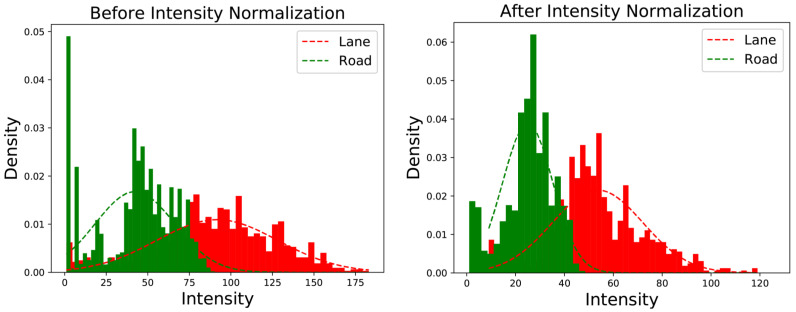
Histogram statistics before (**left**) and after (**right**) the incidence normalization. The red and green columns represent the statistical results of the lane and the road areas, respectively. Gaussian curves were used to fit their mathematical distributions. The smaller overlapping area and the sharper distribution indicate a better result after normalization.

As shown in Figure 9, the histogram indicates that after normalization, the intensity distribution of the same object is more concentrated, which implies a higher consistency after incidence normalization.

#### 4.1.2. Quality of Artificial Intensity Maps

$I^{atf}$ is the artificial intensity map that is collected theoretically for the current frame, so $I^{atf}$ and I should follow a similar distribution and material discrimination. Since it is difficult to compare them from the picture, we used two quantitative results to evaluate the similarity between the artificial intensity and the original raw intensity. In each evaluation, the road surface areas and the lane areas were manually selected for computing the distributions.

If one object’s intensity distribution is assumed as Gaussian distributions, the indicator $R_{V}$ proposed in [32] can be employed to assess the similarity of the intensity distribution for the same object *V* in different intensity maps and is written below:

(13) $$R_{V}=\frac{\sigma_{V\in I^{eva}}}{\mu_{V\in I^{eva}}}/\frac{\sigma_{V\in I}}{\mu_{V\in I}},$$

where μ and σ denote the mean and standard deviation of the selected map regions, and $I^{eva}$ is the intensity map under evaluation. As shown in Table 1, the $R_{V}$ between I and $I^{atf}$ is almost equal to one, which indicates that the results reserve the same pattern of the intensity distribution for the same object.

In order to prove that the artificial intensity retains the distribution difference between different objects, the overlapping coefficient ρ [49] defined below can be used to measure the similarity between different Gaussian distributions from different objects,

(14) $$\rho =1-F_{1}\left(i\right)+F_{2}\left(i\right),$$

where *i* is the intersection between two probability density functions, and F(·) represents the cumulative distribution functions. Therefore, the smaller the ρ value, the higher the discrimination between different materials will be. As shown in Table 2, the ρ of I and $I^{atf}$ are almost equal to each other. The result indicates that, for different objects, I and $I^{atf}$ have similar distributions, implying that the value of $I^{atf}$ is a reasonable synthetic measurement.

### 4.2. Comparison of Intensity Completion

Most methods only focus on LiDAR depth completion, and there are no Lidar intensity completion methods with open-source code available. Therefore, several methods for LiDAR depth completion that can work on a single input type will be evaluated on the LiDAR intensity dataset. Their intensity completion results are then used in the comparison experiment.

For comparison, results were obtained using the following state-of-the-art methods: Sparse-to-dense [15], SparseConvs [19], nConv-CNN [39], and pNCNN [50]. In addition, the reported results of IP-Basic [51] are also included in the comparison. Sparse-to-dense, SparseConvs, nConv-CNN, and pNCNN represent the most advanced methods using learning-based techniques, while IP-Basic leverages non-learning models.

**Figure 10:**
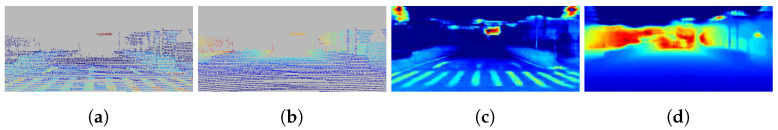
Input (**a**,**b**) and output (**c**,**d**). The proposed completion system takes the sparse depth and intensity from a LiDAR sensor as input (row 1) to obtain the dense completion result (row 2). Each pixel in (**a**,**b**) is enlarged five times to increase the visualization: (**a**) sparse input intensity; (**b**) sparse input depth; (**c**) intensity completion; (**d**) depth completion.

**Figure 11:**
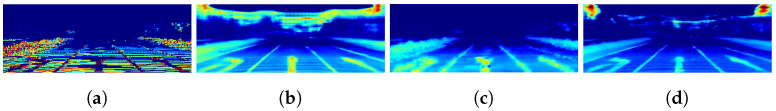
Comparison of intensity completion. Colder color in the intensity map indicates weaker strength: (**a**) Ip-Basic [51]; (**b**) sparse-to-dense [15]; (**c**) pNCNN [50]; (**d**) ours.

As shown in Figure 10, using the proposed dataset, the LiDAR-Net can use spare input to provide dense results. The completion results are satisfactory, except in the area where the original information is missing. A qualitative comparison is shown in Figure 11. Ip-Basic almost failed in Scene 2, and the sparse-to-dense method cannot provide satisfactory results. For pNCNN, although the prediction result is correct around the corner, the edges of the lane on the ground are blurry. Hence, the proposed method outperformed the rest in the completion task while preserving sharper textures.

The quantitative results are shown in Table 3. If the method cannot complete the training, the results are marked with an ‘x’. The results indicate that the proposed method generates the best performance. The reason is that most methods are dedicated to the KITTI completion dataset. In the KITTI dataset, the input depth maps are denser. In contrast, our dataset provides more spare data since many fields are beyond the range of the LiDAR sensors due to the spacious road environment. This sparse dataset makes the completion task more challenging. Moreover, the spatial characteristics of the depth maps used in some methods are invalid for the intensity completion task. As shown in the table, the performance of the learning-based methods is close. The possible reason is that the data, such as the lane lines, account for a small percentage of the overall data. Therefore, the good completion of these areas cannot impact the metric a lot, even though the completion results of the proposed method are clearly better than other methods, as shown in the qualitative comparison.

### 4.3. Completion Ablation Experiments

To verify the architecture of the proposed method, some detailed ablation studies are conducted in this section. There are two ablation experiments to evaluate the effectiveness of intensity–depth fusion and show the necessity of incidence normalization. The first experiment will verify the complementarity between depth and intensity. The second will confirm the benefit of the inverse network with the additional supervision.

#### 4.3.1. Effectiveness of Intensity–Depth Fusion

Unlike the depth completion schemes, the proposed method uses both the depth and intensity data from a single LiDAR to achieve intensity completion jointly. To verify the complementarity of depth and intensity, the model in [15], which is also a U-like network using only intensity information $I^{atf}$, serves as the baseline of the single-input model.

**Figure 12:**
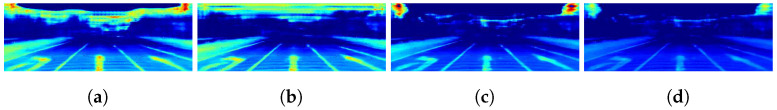
Qualitative analysis of ablation study: (**a**) the intensity map completed by the single input model supervised by $I^{atf}$; (**b**) the completion result from using only the proposed completion backbone network supervised by $I^{atf}$ and $D^{den}$; (**c**,**d**) are $\hat{I}$ and ${\hat{I}}^{norm}$ completed by LiDAR-Net supervised by $I^{atf}$, $D^{den}$, and $I^{norm}$; (**a**) $\hat{I}$ from onlyI ($I^{atf}$); (**b**) $\hat{I}$ from DI-to-DI ($I^{atf}$+$D^{den}$); (**c**) $\hat{I}$ from LiDAR-Net ($I^{atf}$+$D^{den}$+$I^{norm}$); (**d**) ${\hat{I}}^{norm}$ from LiDAR-Net ($I^{atf}$+$D^{den}$+$I^{norm}$).

As depicted in Figure 12a and Table 4, the results from the method using only intensity show that when the information source includes only sparse intensity maps, the completion result has ripples, and the intensity of the lane with the same material is inconsistent. Corresponding to this phenomenon, the fusion of depth improves the consistency of intensity, as shown in Figure 12b. The reason may be that the geometry information can reduce the ripple effect. Therefore, the correlation between depth and intensity learned by the network, especially in those scenes where the intensity value is regularly distributed such as the road surface, can help achieve better completion. Unfortunately, the addition of depth information does not entirely solve the ripple issue due to the intrinsic ripple distribution in the depth and intensity ground truth, as shown in Figure 8.

#### 4.3.2. Effectiveness of Supervision with Normalized Intensity

The LiDAR-Net proposed in Section 3.2 is supervised by $I^{norm}$ at the end of the decoder and by $I^{atf}$ at the end of the inverse network. To verify the effectiveness of the inverse normalization network, we also present the experiment results on DI-to-DI, which is directly supervised by $I^{atf}$ at the end of the decoder but without the inverse normalization network.

As shown in Table 4, based on the DI-to-DI network, LiDAR-Net further enhances the performance of the completion with only a few extra parameters.

Figure 12c shows that with the supervision aided by the densified normalized intensity $I^{norm}$, the ripple effect mentioned above is less evident than the results of DI-to-DI network. Meanwhile, as shown in Figure 12d, the predicted ${\hat{I}}^{norm}$ is more related to materials and more robust to distance and incidence angle. It indicates that the additional material information from $I^{norm}$ enables the network to learn possible distributions of intensity through the correlation between depth and reflectivity, even in the ripple area where the supervision information is unavailable.

**Figure 13:**
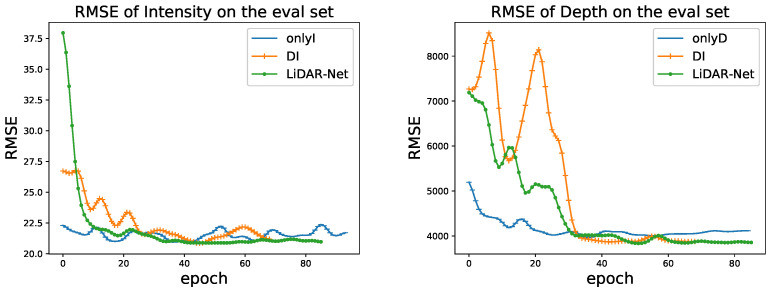
Convergence curves. LiDAR-Net (green) converges on intensity (**left**) faster than DI-to-DI (yellow) and onlyI (blue) and achieves better depth (**right**) completion performance.

We also find that LiDAR-Net converges much faster than DI-to-DI, as shown in Figure 13. The LiDAR-Net improves the completion performance. It also reduces the difficulty of convergence and oscillation in the training process.

The results from the experiments show that the addition of the inverse network leads to a faster and smoother convergence and that $I^{norm}$ serves as an additional supervisor to improve the completion performance.

### 4.4. Comparison of Depth Completion

Since the proposed method has the capability for depth completion, this section will compare the performance of the proposed method with several state-of-the-art depth completion methods [19,39,51] to show the benefit of intensity information. Since the KITTI depth completion benchmark does not provide simultaneously the ground truth of depth and of intensity, we have to test various methods on our dataset.

As shown in the lower part of Table 5, the proposed method, which uses both intensity and depth information, provides the highest accuracy of depth completion according to RMSE. The result proves that using only depth is insufficient to train the network to predict the area where values are missing. In other words, since the input of the proposed dataset is too sparse, the results of the state-of-the-art methods tend to be unsatisfactory. This result explains why many methods seek the assistance of RGB cameras to provide better completion outcomes.

### 4.5. Lane Segmentation

As discussed in the introduction, as an active sensor, LiDAR is not affected by the ambient light and can respond to dramatic lighting variations in autopilot situations. With the proposed method, many modern computer vision methods can be employed, such as object segmentation. To demonstrate the significant advantage of dense LiDAR intensity, we investigate the lane segmentation performance using RGB images versus completed LiDAR intensity under normal and low illumination conditions.

**Figure 14:**
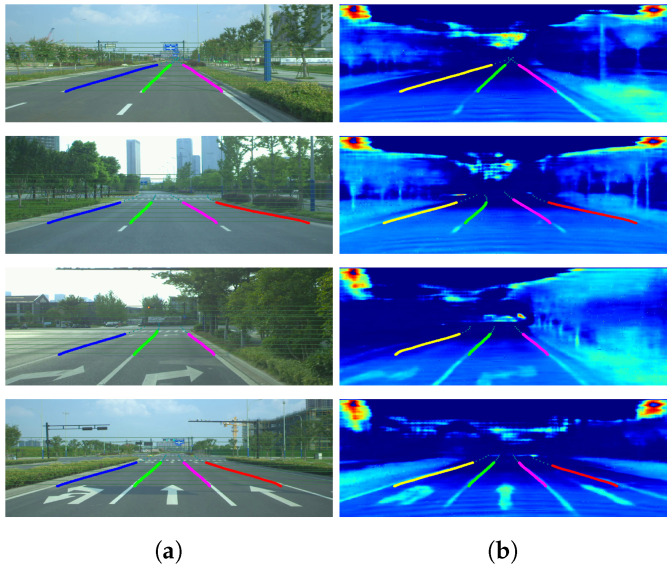
Lane segmentation results in good illumination conditions. Under normal lighting conditions, the dense intensity after completion can reach the same performance as RGB images in lane segmentation. It shows that traditional vision methods can be applied to the LiDAR intensity maps without modification: (**a**) RGB; (**b**) intensity.

**Figure 15:**
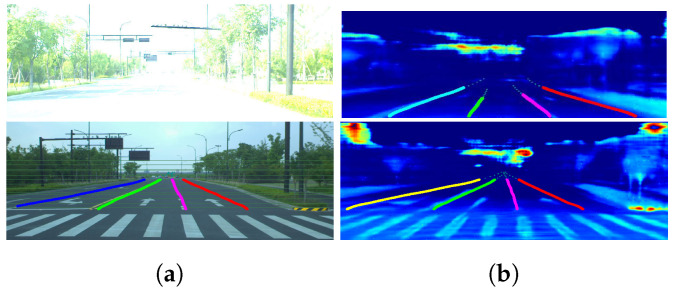
Lane segmentation results in complex illumination conditions. The upper and lower rows are the data under two different illumination conditions. The left and right images are the data from a visible light camera and LiDAR, respectively. Different colored lines indicate the segmentation results of different lanes. It shows that the LiDAR intensity maps have great potential for applications under adverse illumination conditions: (**a**) RGB; (**b**) intensity.

The SCNN [52] is used as a segmentation model. Without any alteration, the results in Table 6 indicate that the dense LiDAR intensity can be used for lane segmentation with comparable performance. The performance of the proposed method can also be proved in Figure 14. Furthermore, it should be noted that, as shown in Figure 15, in complex illumination environments, the lane lines obtained from the completed LiDAR intensity are easy to detect in both scenarios while the RGB images are not usable under certain conditions.

## 5. Conclusions

In this paper, we proposed a LiDAR-Net to achieve joint intensity completion using both the sparse depth and the intensity of the LiDAR sensors. The proposed LiDAR-Net can achieve satisfactory performance in experiments. With the intensity–depth fusion, the proposed method can provide a better performance of the completion. Moreover, a dataset was built for the intensity completion task. In this dataset, the influence of incidence angle and depth is eliminated to obtain more accurate intensity ground truth that can be used for training, which finally helps improve the model’s performance. The supplementary experiment demonstrates that the completed intensity map can be used for lane detection, showing the potential for practical applications. With the validation of lane detection, we hope that the proposed method will appeal to the computer vision community’s interest in LiDAR intensity measurements.

In future work, new BRDF models, such as the Phong [53], Oren–Nayar [54], or the Torrance–Sparrow [55] reflectance model, will be used to replace Lambertian assumption since the intensity is only corrected by the incident angle and may induce the so-called “over-correction effect” [56] on a large incidence angle.

For applications, we will explore the application potential of dense LiDAR intensity in the field of computer vision, such as semantic detection, tracking, and relocalization. In addition to the 2D tasks, the dense intensity completion method can also be extended to 3D vision tasks such as LiDAR-based 3D object detection and classification.

## Figures and Tables

**Table 1:** Intensity consistency of the same object. The closer the value is to 1, the greater the similarity of the same object’s intensity distribution in two intensity maps.

$R_{V}$	I	$I^{\mathrm{norm}}$	$I^{\mathrm{atf}}$
I	1	0.72	0.97

**Table 2:** Intensity consistency of different objects. Similar values indicate that the relative intensity distributions of different objects in the two images are similar.

	I	$I^{\mathrm{norm}}$	$I^{\mathrm{atf}}$
ρ	0.396	0.317	0.407

**Table 3:** Comparison of intensity completion accuracy. The results from state-of-the-art completion algorithms are shown in the bottom part. The best results are shown in bold; ’x’ denotes a failure.

			Scene 1		Scene 2		Mean
			**Intensity**		**Intensity**		**Intensity**
**Method**	**Input**	**Type**	**RMSE**	**MAE**	**RMSE**	**MAE**	**RMSE**
LiDAR-Net (Ours)	intensity + depth	learning	**20.332**	**13.449**	**28.137**	**18.392**	**24.234**
Sparse-to-dense [15]	single intensity	learning	20.676	13.696	28.570	18.767	24.623
SparseConvs [19]	single intensity	learning	25.942	17.460	36.055	27.150	30.999
nConv-CNN [39]	single intensity	learning	x	x	x	x	x
pNCNN [50]	single intensity	learning	22.131	14.911	29.539	19.928	25.835
IP-Basic [51]	single intensity	non-learning	28.725	17.957	56.374	35.784	42.550

**Table 4:** Quantitative analysis of ablation study. The best results are shown in bold. The ablation study indicates that intensity–depth fusion and supervision with normalized intensity can improve the performance.

	Scene 1		Scene 2		Mean
	**Intensity**		**Intensity**		**Intensity**
**Method**	**RMSE**	**MAE**	**RMSE**	**MAE**	**RMSE**
onlyI ($I^{atf}$)	20.676	13.696	28.570	18.767	24.623
DI-to-DI ($I^{atf}$+$D^{den}$)	20.454	13.556	28.237	18.582	24.346
LiDAR-Net ($I^{atf}$+$D^{den}$+$I^{norm}$)	**20.332**	**13.449**	**28.137**	**18.392**	**24.234**

**Table 5:** Comparison of depth completion accuracy. The results from state-of-the-art depth completion algorithms are shown in the bottom part. The best results are shown in bold. We use ‘i’ and ‘d’ to represent Lidar intensity and depth, respectively; ‘x’ denotes a failure.

		Scene 1		Scene 2		Mean
		**Depth [mm]**		**Depth [mm]**		**Depth [mm]**
**Method**	**Input**	**RMSE**	**MAE**	**RMSE**	**MAE**	**RMSE**
LiDAR-Net (Ours)	i + d	**3822.5**	1300.2	**5093.0**	1974.5	**4457.8**
Sparse-to-dense [15]	single d	3900.1	1310.2	5226.3	2165.3	4563.2
SparseConvs [19]	single d	7134.5	3162.3	9486.8	4271.21	8310.7
NConv-CNN [39]	single d	5190.1	1725.2	6534.8	2425.7	5862.4
pNCNN [50]	single d	3956.8	**1110.4**	5104.4	**1816.0**	4530.5
IP-Basic [51]	single d	6645.9	1934.9	8521.6	2159.7	7583.8

**Table 6:** Comparison of lane segmentation results. F1=2×Precision×Recall/(Precision+Recall).

Input Type	Precision	Recall	F1
RGB image from visible cameras	0.957	0.612	0.746
$\hat{I}$ from LiDAR-Net	0.862	0.553	0.674

## Data Availability

Not applicable.
